# Structural biology of DOCK‐family guanine nucleotide exchange factors

**DOI:** 10.1002/1873-3468.14523

**Published:** 2022-11-04

**Authors:** Andreas Boland, Jean‐Francois Côté, David Barford

**Affiliations:** ^1^ Department of Molecular and Cellular Biology University of Geneva Switzerland; ^2^ Montreal Clinical Research Institute (IRCM) Canada; ^3^ Department of Medicine and Department of Biochemistry and Molecular Medicine Université de Montréal Canada; ^4^ MRC Laboratory of Molecular Biology Cambridge UK

**Keywords:** CDC42, DOCK proteins, guanine nucleotide exchange factors, RAC1

## Abstract

DOCK proteins are a family of multi‐domain guanine nucleotide exchange factors (GEFs) that activate the RHO GTPases CDC42 and RAC1, thereby regulating several RHO GTPase‐dependent cellular processes. DOCK proteins are characterized by the catalytic DHR2 domain (DOCK^DHR2^), and a phosphatidylinositol(3,4,5)P_3_‐binding DHR1 domain (DOCK^DHR1^) that targets DOCK proteins to plasma membranes. DOCK‐family GEFs are divided into four subfamilies (A to D) differing in their specificities for CDC42 and RAC1, and the composition of accessory signalling domains. Additionally, the DOCK‐A and DOCK‐B subfamilies are constitutively associated with ELMO proteins that auto‐inhibit DOCK GEF activity. We review structural studies that have provided mechanistic insights into DOCK‐protein functions. These studies revealed how a conserved nucleotide sensor in DOCK^DHR2^ catalyses nucleotide exchange, the basis for how different DOCK proteins activate specifically CDC42 and RAC1, and sometimes both, and how up‐stream regulators relieve the ELMO‐mediated auto‐inhibition. We conclude by presenting a model for full‐length DOCK9 of the DOCK‐D subfamily. The involvement of DOCK GEFs in a range of diseases highlights the importance of gaining structural insights into these proteins to better understand and specifically target them.

## Graphical abstract

This review discusses structural studies that have provided mechanistic insights into the function of DOCK guanine nucleotide exchange factors (GEFs). Representative structures of members of the DOCK family of GEFs in complex with their cognate Rho GTPases are shown (read the review for more details).

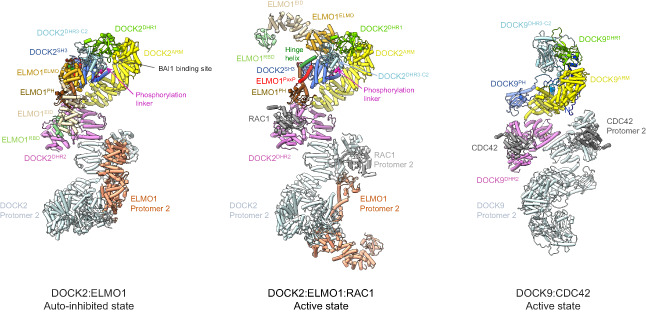

## Abbreviations

**DHR1**, DOCK homology region 1

**DHR2**, DOCK homology region 2

**PtdIns(3,4,5)P**_**3**_, phosphatidylinositol(3,4,5)P_3_

Rho GTPases are conserved regulators of cell motility, polarity, adhesion, cytoskeletal organization, proliferation and apoptosis that stimulate signalling when in the active GTP‐bound state [1, 2, 3]. Activation of these biomolecular switches is controlled by guanine nucleotide exchange factors (GEFs) that catalyse exchange of GDP for GTP [4, 5, 6]. Genetic studies in model organisms were instrumental in uncovering molecular interactions between *ced‐5* (*Caenorhabditis elegans*) and *myoblast city* (*Drosophila*), orthologues of mammalian DOCK1 and the RAC GTPase, respectively [7, 8]. Building on these observations, the interaction of RAC1 with DOCK1 in human 293T cells was demonstrated [9]. Three independent studies reported that DOCK‐family proteins, notably DOCK1 and DOCK9, are GEFs for RAC and CDC42 GTPases, respectively [10, 11, 12]. These efforts revealed a GEF family for RHO proteins lacking the Dbl Homology/Pleckstrin Homology (DH/PH) module present in the large family of Dbl GEFs. It is now established that the DOCK GEFs activate RAC and CDC42 to control cell migration, morphogenesis and phagocytosis, and have been implicated as important components of tumour‐cell movement and invasion [13, 14, 15, 16, 17, 18]. DOCK1, which cooperates with oncogenes such as K‐RAS and HER2, has been extensively targeted in drug discovery efforts. A DOCK1‐specific inhibitor blocks K‐RAS‐induced RAC1 activation, and macropinocytosis, thereby decreasing cell proliferation and invasion [19]. Similarly, an inhibitor that prevents RAC activation by DOCK1/2/5 blunted HER2‐mediated invasion [20]. These examples represent a range of diseases involving DOCK GEFs [21, 22], and highlight the importance of gaining structural insights into these proteins to better understand, and specifically target them.

**Fig. 1:**
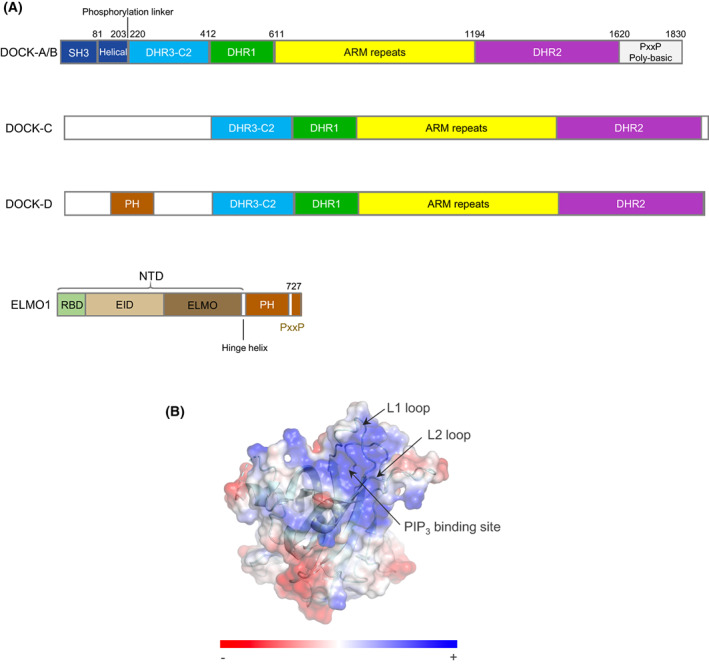
DOCK proteins belong to four subfamilies. (A) Schematic of DOCK subfamily architecture. Subfamilies: DOCK‐A (DOCK1,2,5), DOCK‐B (DOCK3,4), DOCK‐C (DOCK6‐8) and DOCK‐D (DOCK9‐11). (B) Surface representation of the DOCK1^DHR1^ domain. DOCK1^DHR1^ adopts a C2‐like domain architecture that binds PtdIns(3,4,5)P_3_ by means of a positively charged pocket defined by surface loops L1 and L2 [26] (PDB: 3L4C). Figures were generated using chimerax [65].

In humans, DOCK proteins are organized into four subfamilies, characterized by their differing specificities for RAC and CDC42, and associated regulatory domains and subunits (Fig. 1A). DOCK‐A and ‐B subfamilies activate RAC, the DOCK‐D subfamily is mainly specific for CDC42, whereas the DOCK‐C subfamily and DOCK10 of the DOCK‐D subfamily have dual specificity for RAC and CDC42. All contain a GEF catalytic domain (DOCK homology region 2: DHR2) situated within the C terminus and a second conserved region, the DHR1 domain. The DHR1 domains of DOCK1 and DOCK2 interact with the phosphatidylinositol(3,4,5)P_3_ (PtdIns(3,4,5)P_3_) head group to mediate membrane localization and DOCK‐catalysed activation of membrane‐associated RAC [23, 24, 25, 26, 27]. In this review, we mostly focus on the structure and mechanisms of human DOCK‐family proteins. Other DOCK proteins were recently reviewed in [18, 28].

Structural studies on DOCK‐family proteins initiated over a decade ago have recently culminated in structures of the full‐length DOCK2:ELMO1 complex [29] and DOCK5:ELMO1 complex [30]. Advances in structural biology techniques, specifically single‐particle cryo‐electron microscopy, and methods to overexpress DOCK:ELMO complexes using the heterologous insect cell/baculovirus expression system [31], aided structural analysis of these large and dynamic complexes. The aims of structural investigations were multiple: understanding (a) the basis of PtdIns(3,4,5)P_3_ recognition by the DHR1 domain, (b) the basis for GEF catalytic activity by DHR2, (c) the role of the ELMO subunit in the DOCK‐A and DOCK‐B subfamilies, (d) the overall architecture of DOCK proteins and DOCK:ELMO complexes, (e) the basis of the specificity for CDC42 and RAC GTPases, (f) regulatory mechanisms and (g) finally comparison to the larger and well‐studied Dbl‐family GEFs.

Early structural studies of DOCK proteins focussed on the conserved DHR1 and DHR2 domains that are common to all DOCK proteins. The structure of the DHR1 domain of DOCK1 revealed a type II C2‐like homology domain with specificity for PtdIns(3,4,5)P_3_ and PtdIns(4,5)P_2_ [26]. Modelling and mutagenesis indicated that the lipid phospho‐inositol head group binds to a basic pocket on its upper surface (Fig. 1B). Disruption of this pocket ablated DOCK1‐mediated cell polarization, and its structural conservation within the DOCK family suggested a common mechanism of phosphoinositide binding in all DOCK proteins [26]. A recent study of DOCK8^DHR1^ revealed a preference for PtdIns(4,5)P_2_ over PtdIns(3,4,5)P_3_ due to residues in loop 1 colliding with a phosphate group at position 3 of PtdIns(3,4,5)P_3_ [32].

## DOCK proteins catalyse nucleotide exchange using an invariant nucleotide sensor in DOCK^DHR2^

**Fig. 2:**
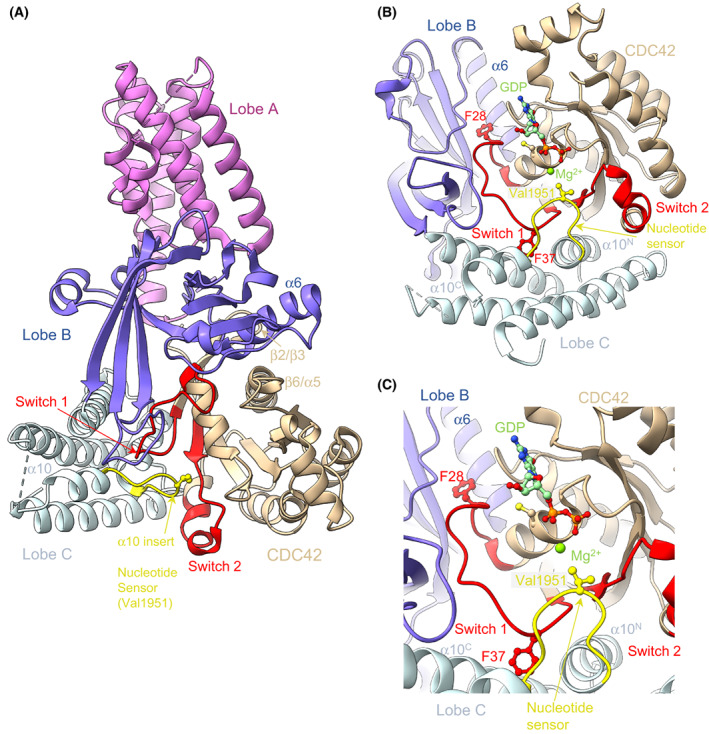
Structure of DOCK9^DHR2^:CDC42. DOCK proteins catalyse nucleotide exchange through a nucleotide sensor. (A) The catalytic DHR2 domain is composed of three lobes, with lobes B and C contacting the RHO GTPase CDC42 directly. (B, C) Details of the nucleotide sensor in the α10 helix of lobe C clamping open switch 1 of CDC42 and protruding into the GDP‐Mg^2+^‐binding site. This directly occludes Mg^2+^ binding. GDP‐Mg^2+^ is modelled into the nucleotide‐free CDC42 based on structures of DOCK9^DHR2^:CDC42:GDP and DOCK9^DHR2^:CDC42:GTP‐Mg^2+^. Figure based on [33] (PDB: 2WM9, 2WMN, 2WMO).

**Fig. 3:**
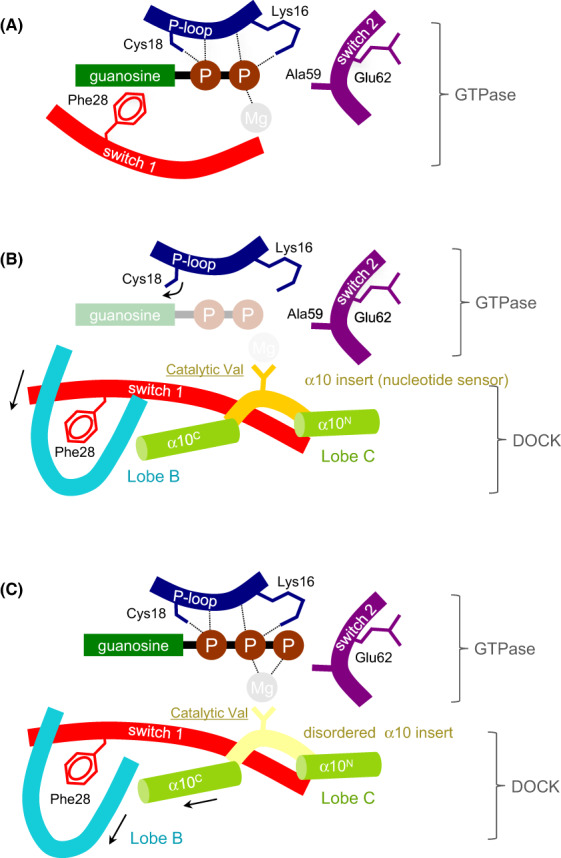
Schematic of the DOCK GEF catalytic mechanism. (A) GDP bound to the nucleotide‐binding site of Rho GTPase. (B) DOCK^DHR2^‐mediated release of GDP occurs *via* the motion of Cys18 and Phe28, disrupting contacts to GDP and exclusion of Mg^2+^ mediated by the catalytic valine (Val1951 of human DOCK9). (C) The binding of GTP‐Mg^2+^ to DOCK^DHR2^‐GTPase promotes conformational changes that trigger the discharge of the activated GTPase. Adapted from [33]. Numbered residues refer to CDC42.

**Fig. 4:**
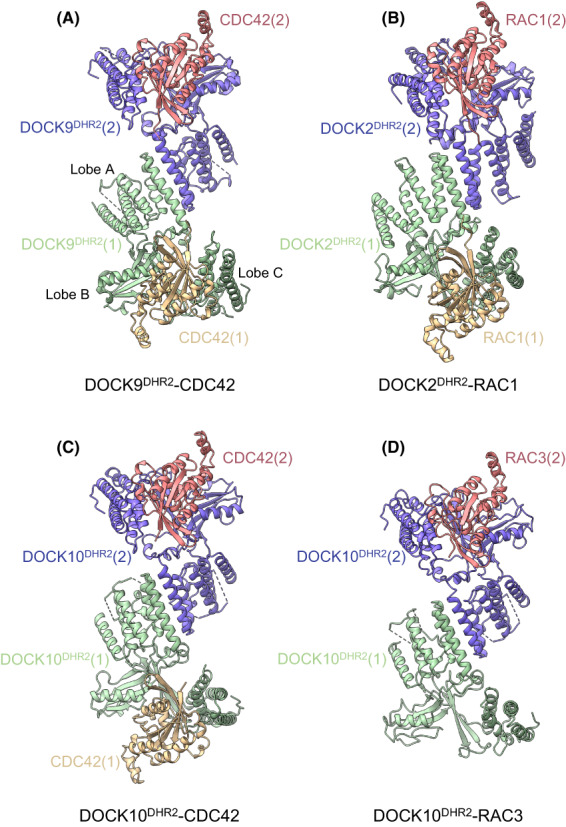
DHR2 domains of DOCK proteins form conserved homodimeric assemblies. Gallery of DOCK^DHR2^:GTPase complexes. (A) DOCK9^DHR2^:CDC42 ([33] (PDB: 2WM9)), (B) DOCK2^DHR2^:RAC1 ([37] (PDB: 2YIN)), (C) DOCK10^DHR2^:CDC42 ([43] (PDB: 6TKY)) and (D) DOCK10^DHR2^:RAC3 ([43] (PDB: 6TM1)). Numbers in parentheses refer to the two protomers of the DOCK dimers.

**Fig. 5:**
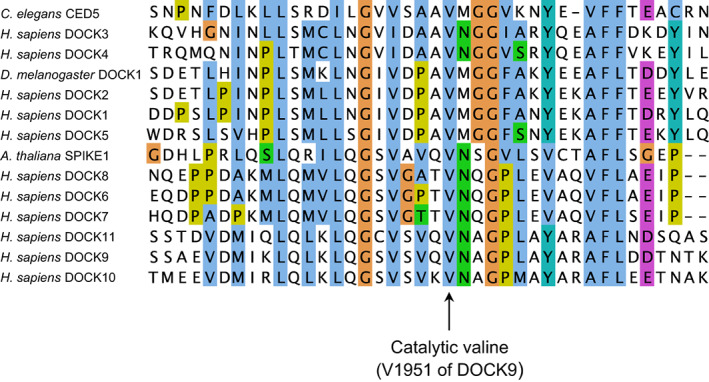
The catalytic valine of the nucleotide sensor is invariant in the DOCK GEF family. Multiple‐sequence alignment of human DOCK proteins, *Caenorhabditis elegans* CED‐5, *Drosophila melanogaster* DOCK1 and *Arabidopsis thaliana* SPIKE1. Figure generated using jalview [66]. The catalytic valine (Val1951 of *Homo sapiens* DOCK9) of the nucleotide sensor is invariant.

Insights into the catalytic mechanism of DOCK‐mediated GEF activity came from structures of the DOCK9 DHR2 domain (DOCK9^DHR2^) in complex with CDC42, both nucleotide free and with either GDP or GTP‐Mg^2+^ [33] (Fig. 2A). A schematic representation is shown in Fig. 3. These structures revealed the conserved DHR2 domain to be organized into three distinct lobes (named A, B and C), with the catalytic segment comprised mainly of lobes B and C. This was later supported by studies of DOCK7 [34] and DOCK8 [35], showing that lobes B and C are sufficient for GEF activity. Lobe A (a TPR‐like fold) stabilizes lobe B, explaining why including this region of DHR2 enhances catalytic activity. Lobe A also creates the DOCK–dimer interface (Fig. 4A). Disruption of the DOCK9^DHR2^ dimer interface decreased catalytic efficiency by twofold [33]. Lobe B adopts an unusual architecture of two antiparallel β‐sheets disposed in a loosely packed orthogonal arrangement, whereas lobe C comprises a four‐helix bundle (Fig. 2A). Helix α10 of lobe C, the most conserved region of DHR2 domains, is interrupted by a seven‐residue loop, the α10 insert, that divides the helix into α10^N^ and α10^C^ (Figs 2B,C and 5).

The mechanism of nucleotide exchange catalysed by DOCKs shares many features of other GEF mechanisms, namely destabilizing the nucleotide and Mg^2+^‐binding sites through structural rearrangements of the GTPase catalytic site (Figs 2B,C and 3). In the DOCK9^DHR2^:CDC42 complex, lobes B and C of DOCK9^DHR2^ tightly embrace the GTPase (Fig. 2A). Interactions of CDC42 with DOCK9 are dominated by switch 1 and augmented through interactions with switch 2, the β2/β3 hairpin and β6/α5 loop of CDC42. When in complex with DOCK9^DHR2^, switch 1 undergoes a conformational change that exposes CDC42's nucleotide‐binding site. In contrast to many other GEFs, switch 2 remains unchanged. Extensive interactions between the two proteins stabilize the displaced conformation of switch 1. The α10 insert containing the invariant and essential Val1951 residue [33] (Fig. 5) clamps open switch 1, allowing the aliphatic side chain of Val1951 to project into the GTPase nucleotide‐binding site. Val1951 functions as a nucleotide sensor to mediate nucleotide release and discharge of the activated (GTP‐Mg^2+^ bound) GTPase from DOCK^DHR2^.

As revealed by structural analysis of a complex of DOCK9^DHR2^:CDC42 with GDP [33], the Mg^2+^‐binding pocket is occluded by the Val1951 side chain of the nucleotide sensor. Comparing the nucleotide‐free and GDP‐bound DOCK9^DHR2^:CDC42 complexes defined the molecular basis for the DOCK9^DHR2^‐mediated decrease in nucleotide affinity. First, the conformational transition of switch 1 on binding DOCK9^DHR2^ exposes the nucleotide‐binding site. Displacement of the CDC42 switch 1 residue Phe28 disrupts the conserved aromatic guanine base interaction important in guanine nucleotide binding [36] (Fig. 2B). Second, by intruding into the GTPase nucleotide‐binding site, Val1951 directly occludes the nucleotide‐coordinated Mg^2+^. Because magnesium enhances nucleotide affinity by neutralizing the negatively charged phosphate groups, its exclusion profoundly reduces nucleotide affinity [4]. The key role of Val1951 was confirmed by the observation that substitution with an Ala residue severely abrogated catalytic activity [33]. In DOCK9^DHR2^, but not DOCK2^DHR2^ [37], the movement of switch 1 is linked to the rotation of the GTPase P‐loop Cys18 thiol group that disrupts a hydrogen bond with the nucleotide α‐phosphate. This mechanism of magnesium exclusion by a direct effect of a residue within the catalytic domain differs from that found in other GEFs where displacement of the conserved switch 2 Ala residue of the GTPase (Ala59 in RAS and RHO family GTPases) sterically interferes with Mg^2+^ binding [38, 39, 40, 41]. In these GEFs, the motion of switch 2 also creates a salt bridge between Glu62 of switch 2 and Lys16 of the P loop, conferring charge repulsion on the nucleotide's phosphate groups and disrupting their favourable interaction with Lys16. In contrast, DOCK9^DHR2^ does not alter the positions of either switch 2 or Lys16 of CDC42. Thus, unlike Dbl‐family GEFs [42], DOCK GEFs would not use either Ala59 or Glu62 of the RHO GTPase for nucleotide exchange. Consistent with this notion, substituting Gly for Ala59 or Ala for Glu62 of CDC42 did not diminish the nucleotide exchange activity of DOCK9^DHR2^ towards the GTPase [33].

Activation of CDC42 requires binding of GTP‐Mg^2+^, and subsequent discharge from DOCK9. A structure of DOCK9^DHR2^:CDC42:GTP‐Mg^2+^ indicated that with GTP‐Mg^2+^ bound to CDC42 in the DOCK9^DHR2^:CDC42 complex, the nucleotide sensor insert is disordered, removing the clamp on switch 1. Conformational changes elsewhere in DOCK9^DHR2^ involve conformational changes within lobe B. This reorganization of the complex disrupts DOCK9^DHR2^ contacts to switch 1 and is indicative of the disengagement of CDC42 from DOCK9^DHR2^ and its transition to an enhanced affinity for nucleotide. The motion of α6 of lobe B enlarges the binding pocket for Phe28 (Fig. 2B,C), partially exposing Phe28, whereas rotation of the Cys18 thiol group of CDC42 to hydrogen bond with the α‐phosphate of GTP increases GTP affinity. Thus, activation of CDC42 is detected by the presence of Mg^2+^ tightly bound to GTP. This triggers displacement of the α10 insert and propagation of conformational changes to the DOCK9^DHR2^:CDC42 interface. Because GDP does not induce equivalent conformational changes, GTP may specifically promote the discharge of the GTP‐bound CDC42 from DOCK9^DHR2^ to complete the catalytic exchange reaction. Thus, the α10 insert of DOCK proteins acts as a sensor of the GDP‐ and GTP‐bound states of CDC42.

Subsequent studies on the RAC‐specific GEF, DOCK2^DHR2^ [37] and dual‐specificity DOCKs, DOCK7 [34] and DOCK10 [43] have confirmed the evolutionary conservation of the nucleotide exchange mechanism mediated by the nucleotide sensor residue (Val1951 of DOCK9) that is invariant across the DOCK family in all species (Fig. 5).

## Multiple factors determine CDC42 and RAC specificity

**Fig. 6:**
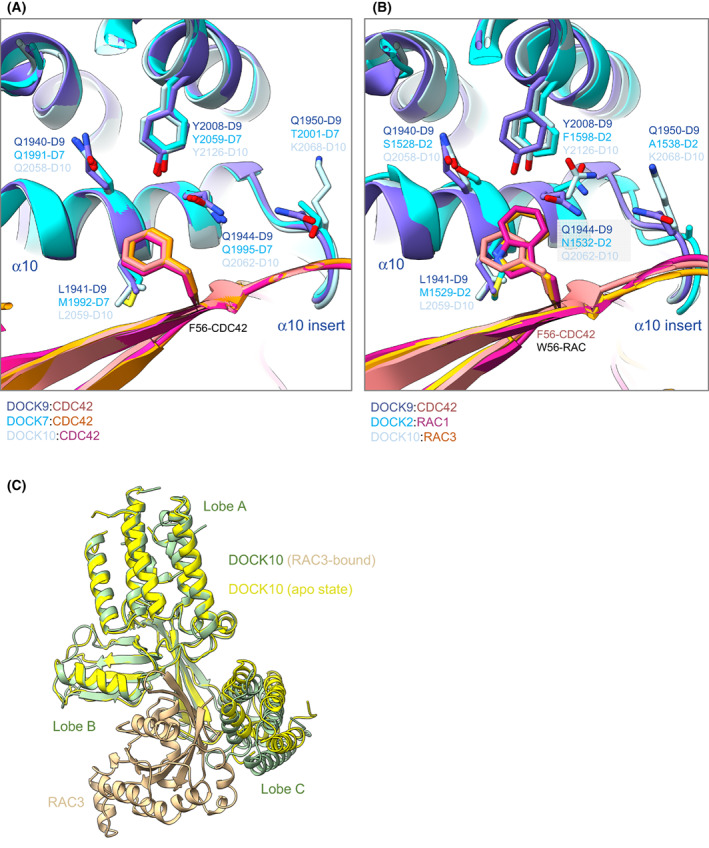
DOCK specificities for CDC42 and RAC through position 56 of CDC42 and RAC. (A) Superimposition of DOCK9^DHR2^:CDC42 ([33] (PDB: 2WM9)), DOCK10^DHR2^:CDC42 ([43] (PDB: 6TKY)) and DOCK7^DHR2^:CDC42 ([34] (PDB: 6AJ4). (B) The larger Trp56 in RAC is accommodated by the smaller and rotated Asn1532 side chain in DOCK2 and the rotation of Gln2062 in DOCK10. Superimposition of DOCK9^DHR2^:CDC42 ([33] (PDB: 2WM9)), DOCK2^DHR2^:RAC1 ([37] (PDB: 2YIN)) and DOCK10^DHR2^:RAC3 ([43] (PDB: 6TM1)). (C) Superimposition of apo‐DOCK10^DHR2^ onto lobes A and B of DOCK10^DHR2^ of the DOCK10^DHR2^:RAC3 complex shows the relative shift of lobe C to a more open state in apo‐DOCK.

Understanding the molecular basis of DOCK specificity for a specific RHO GTPase was made possible by a comparative analysis of the DOCK:RHO GTPase interface of the CDC42‐ and RAC‐specific DOCKs, DOCK9 and DOCK2, respectively [33, 37] (Figs 4 and 6A,B). The DOCK^DHR2^‐GTPase contact residues that differ between RAC1 and CDC42 comprise only six residues located among β1, switch 1 and β3, and are confined to the N‐terminal 56 residues of the GTPase. These six divergent residues, and associated differences in the conformation of switch 1, are responsible for conferring the majority of DOCK‐GTPase specificity. These involve specific hydrogen bonds only possible with the specific DOCK‐GTPase pairing, and van der Waals compatibility (as discussed in [37]).

DOCK9^DHR2^ cannot bind RAC because of the steric clashes caused by the exchange of Val33 and Phe56 in CDC42 with the bulkier Ile and Trp residues, respectively, in RAC. The RHO GTPase‐Phe/Trp56‐binding pocket in DOCK^DHR2^ domains is likely an important specificity determinant. In the DOCK9^DHR2^‐CDC42 complex, the Phe56 side chain of CDC42 is tightly packed by Tyr2008, Gln1944 and Leu1941, whereas in DOCK2^DHR2^, to accommodate Trp56 of RAC, the equivalent residues are the less bulky Phe1598, Asn1532 and Met1529 (Fig. 6B).

Exchanging the six residues in CDC42 for those in RAC, and *vice versa*, indicated that these six residues alone do not account for all the DOCK2^DHR2^:RAC and DOCK9^DHR2^:CDC42 specificity [37]. For example, although the CDC42^6M^ mutant (with the six RAC residues in place of the CDC42 residues) was no longer activated by DOCK9^DHR2^, it was activated by DOCK2^DHR2^ to only 50% of the level of RAC1 stimulation. Likewise, RAC^6M^ could not be activated by DOCK2^DHR2^, and was activated by DOCK9^DHR2^ to 40% of the level of RAC1. This indicated that the variable residues of CDC42 and RAC that directly contact their cognate DOCK^DHR2^ do not solely contribute to specificity. Other variable residues that indirectly affect the conformation of the DOCK^DHR2^‐GTPase interface, specifically switch 1 that differs in conformation between the DOCK2^DHR2^:RAC1 and DOCK9^DHR2^:CDC42 complexes, also contribute. The importance of the residue at position 56 of CDC42 and RAC (Phe/Trp) in conferring specificity with DOCK9^DHR2^ and DOCK2^DHR2^, respectively, was confirmed by the observation that a mutant of RAC1 with Phe substituted for Trp56, was activated to 20% of the level of CDC42 by DOCK9^DHR2^ and had a severely reduced activation by DOCK2^DHR2^ [37].

Further insights into DOCK^DHR2^‐GTPase specificity were obtained from structures of the dual‐specificity DOCKs, DOCK7 and DOCK10, with CDC42 and RAC (Figs 4 and 6A,B). These structures revealed that the DOCK^DHR2^ Met/Leu and Tyr/Phe residues of the GTPase Phe/Trp56‐binding pocket are unlikely to confer CDC42/RAC specificity. The dual specificity DOCKs, DOCK7 and DOCK10, have the Met/Tyr and Leu/Tyr combinations, respectively, showing that both a Leu and Met (equivalent to Leu1941 of DOCK9) and Phe and Tyr (equivalent to Tyr2008 of DOCK9) are compatible with activation of both CDC42 and RAC. A likely important position is Gln1944 of DOCK9. Although DOCK7 (Gln1995) and DOCK10 (Gln2062) share this conserved Gln with DOCK9, in the latter, Gln forms a hydrogen bond with Gln1950 (of the α10 insert), restricting the rotamer conformation of Gln1944, thereby constricting the size of the Phe56‐binding pocket. This Gln‐Gln pairing is not conserved in DOCK7 and DOCK10 (Thr and Lys replace Gln1950, respectively), allowing the equivalent of Gln1944 to adopt side‐chain rotamer conformations compatible with a Trp56 residue (Fig. 6A). In support of this suggestion, DOCK11, a CDC42‐specific GEF, conserves a Gln residue equivalent to Gln1950 of DOCK9. Another factor contributing to DOCK10^DHR2^ activity towards RAC are unique contacts between the GEF and RAC1 not present in DOCK9 (and likely DOCK11) that would enhance the affinity of DOCK10 for RAC1. For example, Arg2056 of DOCK10 forms a hydrogen bond to Asn52 in RAC1 (Thr in CDC42), a contact not available to DOCK9 and DOCK11 (Lys and Gln, respectively). Other factors implicated in contributing to specificity are detailed in [34, 37, 43, 44].

A complex of DOCK10^DHR2^ with RAC3 is the only structure of a dual‐specificity DOCK GEF in complex with RAC [43]. Surprisingly the crystal structure, confirmed by ITC and SEC‐MALS data, revealed an asymmetric arrangement of one RAC3 molecule bound to a DOCK10^DHR2^ dimer, whereas the DOCK2^DHR2^:CDC42 structure showed the expected 2 : 2 stoichiometry (Figs 4C,D and 6C). The interaction of RAC3 with DOCK10^DHR2^ is very similar to the DOCK10^DHR2^:CDC42 complex, with a small rotation of the A and B lobes relative to lobe C. The unoccupied apo‐DOCK10^DHR2^ protomer in contrast revealed a marked rotation of lobe C away from lobes A and B that form a rigid body (Fig. 6C). This state likely presents the more open conformation of DOCK^DHR2^ lobes A and B relative to C to accommodate the initial engagement of a nucleotide‐bound RHO GTPase, prior to DOCK^DHR2^‐induced opening of switch 1 and release of nucleotide Mg^2+^.

The molecular mechanism of the negative cooperativity of DOCK10^DHR2^ to RAC3 binding, and how this is specific to RAC3, is not clear. However, it may contribute to the capacity of DOCK10 to activate RAC, and perhaps the molecular pathway allowing DOCK10 to bind RAC with negative cooperativity is not available to DOCK9 and DOCK11, thus explaining why, within the DOCK‐D subfamily, only DOCK10 possesses dual specificity for CDC42 and RAC. The activity of DOCK10^DHR2^ *in vitro* for both CDC42 and RAC [43] is comparable to the GEF activity of DOCK9^DHR2^ for CDC42 [33] (approximately 50‐ to 400‐fold rate enhancement relative to spontaneous nucleotide exchange rates), and substantially higher than the reported GEF activity of DOCK7^DHR2^ *in vitro* (two‐ to three‐fold enhancement) [34]. Since full‐length DOCK10 functions as a dual‐specificity GEF *in vivo*, [45, 46, 47, 48], the dual specificity of DOCK10^DHR2^ *in vitro* is not simply a feature of the isolated DHR2 domain. Likewise, the dual specificity of full‐length DOCK7 has been demonstrated *in vivo* [47, 48].

## Architecture of DOCK:ELMO complexes suggests mechanisms of regulation

**Fig. 7:**
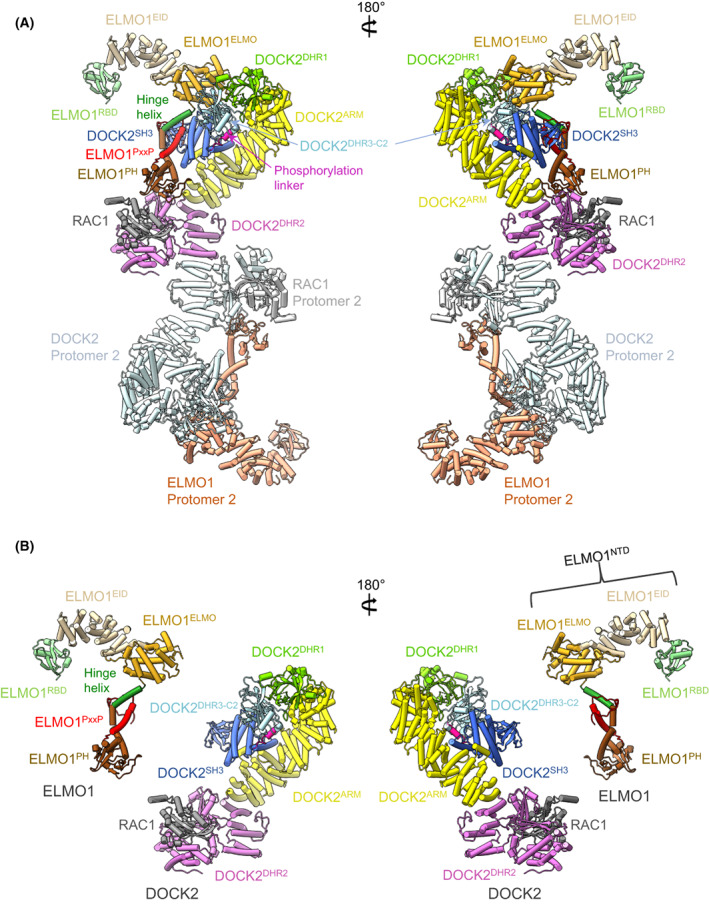
Cryo‐EM structure of DOCK2:ELMO1:RAC1. (A) Orthogonal views of the complex. (B) As in (A), but DOCK2:RAC1 and ELMO1 are separated for clarity and only one DOCK2:ELMO1:RAC protomer is shown. Figure based on [29] (PDB: 6TGC).

**Fig. 8:**
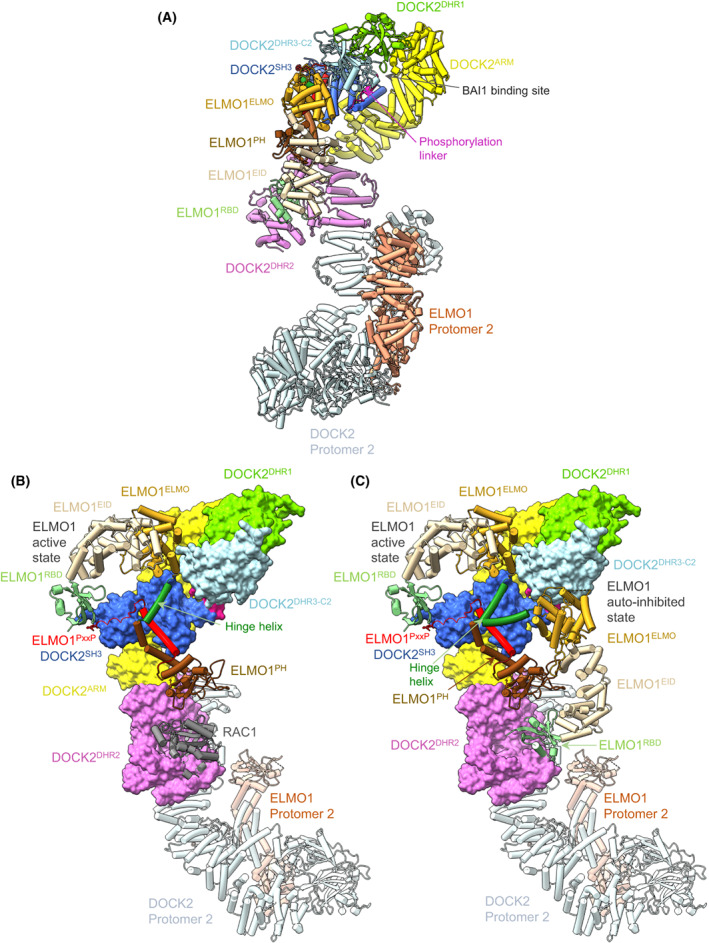
The binary DOCK2:ELMO1 complex adopts an auto‐inhibited conformation. (A) Cryo‐EM structure of binary DOCK2:ELMO1 with ELMO1 adopting a closed auto‐inhibited state. (B, C) Comparison between the open and closed conformation of ELMO1 explains steric inhibition of RAC1 binding to DOCK2^DHR2^. (B) DOCK2:ELMO1:RAC1 with DOCK2 shown as a molecular surface, ELMO1 in the open, active conformation and RAC1 as a cartoon. (C) DOCK2:ELMO1:RAC1 with DOCK2 shown as a molecular surface, ELMO1 shown in the open (up) active conformation (ternary complex) and in the closed (down) auto‐inhibited state (binary complex), RAC1 and DOCK2 Protomer 2 as a cartoon. Rotation of ELMO1^NTD^ about the hinge helix allows interconversion between open active and closed auto‐inhibited. Figure based on [29] (PDB: 6TGB, 6TGC).

Within the past 2 years, the structures of full‐length DOCK2:ELMO1 (Fig. 7) [29] and DOCK5:ELMO1 [30] have been determined using cryo‐EM, a technique ideally suited to the large size and conformational flexibility of these complexes. The DOCK2:ELMO1 binary and DOCK2:ELMO1:RAC1 ternary structures were recently updated and guided by alphafold2 predictions [49] that allowed side‐chain modelling of regions of the molecule that were resolved at lower resolution in the cryo‐EM maps (A.B. and D.B., unpublished; PDB: 6TGB, 6TGC). Comparison of the binary DOCK2:ELMO1 complex with the ternary DOCK2:ELMO1:RAC1 complex revealed an auto‐inhibited state of apo‐DOCK2:ELMO1, and possible mechanisms for its activation by upstream effectors (Figs 7 and 8) [29].

The structures of the DOCK2:ELMO1:RAC1 and DOCK5:ELMO1:RAC1 dimeric complexes are essentially similar [29, 30]. Due to flexibility, only ELMO1^PH^ is visible in the cryo‐EM maps of DOCK5:ELMO1:RAC1 [30]. The dimeric DOCK2:ELMO1:RAC1 complex adopts an elongated ‘S'‐like shape with a pseudo‐twofold symmetry, measuring 320 and 65 Å in the longest and shortest dimensions respectively (Fig. 7). Both the DOCK2 and ELMO1 subunits are assembled from a series of modular functional domains separated mainly by ARM repeat‐like α‐solenoid segments. In the complex, these two subunits are arranged in a roughly parallel manner, but owing to DOCK2 adopting a hook‐like structure, its N‐terminal DOCK2^SH3^ domain is positioned to interact with the C‐terminal poly‐proline motif of ELMO1 (Fig. 7). Located at the centre of the DOCK2:ELMO1:RAC1 tertiary complex is the DOCK2^DHR2^ domain, dimerized through its A lobe, and associated with nucleotide‐free RAC1 through its B and C lobes. DOCK2 and ELMO1 interact through the N‐terminal DOCK2^SH3^ domain engaging the C‐terminal poly‐proline sequence of ELMO1, together with an intermolecular α‐helical bundle formed from the coalescence of three α‐helices adjacent to DOCK2^SH3^ and two α‐helices adjacent to ELMO1^PH^, a structural module of DOCK2:ELMO1 determined previously [50]. The cryo‐EM structures revealed a C2 domain connecting this region of DOCK2 and DOCK5 with DOCK^DHR1^. Since DOCK^DHR1^ also adopts a C2‐domain architecture, DOCK^C2^ and DOCK^DHR1^ form a tandem C2 domain fold, with both C2 domains tightly packed. As discussed below, this C2 domain is conserved in the DOCK family from animals to the plant DOCK‐GEF SPIK1. As proposed by Mallery et al. [51], we adopt the term DHR3‐C2.

A long α‐helical solenoid (approximately 20 α‐helices in DOCK2, and conserved within the DOCK family; named DOCK2^ARM^) connects DOCK^DHR1^ with DOCK^DHR2^. The α‐helical solenoid comprises a mixture of ARM/HEAT and TPR‐like motifs. ARM/HEAT repeats form arrays of parallel α‐helices, whereas the α‐helices of TPR motifs are arranged in a more extended anti‐parallel arrangement. The five α‐helices of DOCK^DHR2^ lobe A are TPR‐like. DOCK2^ARM^ forms intramolecular interactions with DOCK2^SH3^ and adjacent α‐helices, mediated in part by a short α‐helix inserted within DOCK2^ARM^ that projects out from the main α‐solenoid. This intramolecular interface suggests that although extended molecules, DOCK2 and DOCK5 may form reasonably rigid structures, a notion supported by the essential similar conformations of DOCK2 in the ternary (RAC1‐bound) and binary complexes, despite structural changes in ELMO1.

In the ternary complex, ELMO1 adopts an elongated, gently curved shape, comprising two distinct structural segments: ELMO1^PH^ together with C‐terminal PxxP motif, and ELMO1^NTD^ (Fig. 7). ELMO1^NTD^ itself is organized into the N‐terminal ELMO1^RBD^, and an α‐solenoid domain. ELMO1^RBD^ adopts a ubiquitin‐like fold and is exposed to solvent, accessible for interactions with RHOG [52] and ARL4A [53]. The α‐solenoid domain is organized into a short ARM/HEAT segment (ELMO^EID^) and a multi‐α‐helical bundle termed the ELMO domain. Importantly, ELMO1^NTD^ and ELMO1^PH^ are connected by a single α‐helix termed the hinge helix. A flexible hinge at the C terminus of the hinge helix allows the mobility of ELMO1^NTD^ to adopt extended and closed conformations.

In an interaction that is important for DOCK2 GEF activity [12, 54, 55, 56, 57], ELMO1^PH^ is positioned in a groove between lobe B of DOCK2^DHR2^ and the nucleotide‐free RAC1, directly interacting with RAC1. The DOCK2:ELMO1 structure, therefore, suggested that ELMO1^PH^ is directly involved in DOCK2 GEF activity by interacting simultaneously with DOCK2^DHR2^ and nucleotide‐free RAC1, thereby stabilizing DOCK2^DHR2^:RAC1 interactions. Consistent with the observed ELMO1^PH^:RAC1 interaction, ELMO1 enhances DOCK2 GEF activity [29]. Based on a unique salt bridge interaction between ELMO1^PH^ and RAC1 (Arg163), it was proposed that the ELMO1^PH^:RAC1 interaction may also contribute towards DOCK‐A subfamily specificity for RAC [30]. In contrast to the PH domains of Dbl‐family GEFs such as Sos1, the non‐canonical ELMO1^PH^ lacks critical phosphoinositide‐binding residues and therefore is not involved in membrane attachment [58].

Cryo‐EM analysis of the binary DOCK2:ELMO1 complex revealed two conformational and functional states (Fig. 8): an open conformation in which ELMO1 adopts an extended conformation as in the ternary DOCK2:ELMO1:RAC1 complex (Fig. 8B), and, in the majority of particles, a closed auto‐inhibited state in which the ELMO^NTD^ has rotated about the flexible hinge helix, shifting ELMO1^NTD^ towards DOCK2^DHR2^, with ELMO1^RBD^ directly contacting the RAC1‐binding site of DOCK2^DHR2^ (Fig. 8A,C). This directly obstructs RAC1 binding to DOCK2^DHR2^, suggesting an auto‐inhibited conformation [29]. In support of this suggestion, deleting ELMO1^NTD^ increased GEF activity by 60% [29]. A related auto‐inhibitory mechanism is likely shared with DOCK5‐ELMO1, however, in this case, removal of ELMO1^NTD^ caused a more substantial 290% increase in GEF activity [30]. It was proposed that ELMO1^NTD^ exhibits a distinct influence on the GEF activities of DOCK5 and DOCK2 [30].

Mapping the binding sites on ELMO1 which interact with a variety of up‐stream signalling proteins suggested mechanisms for activation of the closed auto‐inhibited state of DOCK2:ELMO1 [29]. RHOG in the activated GTP‐bound state interacts with ELMO1^RBD^ [29]. A crystal structure of an RHOG:ELMO2^RBD^ complex revealed unexpectedly that the interface between RHOG and ELMO2^RBD^ comprises both switch 1 and switch 2 regions of the GTPase, in contrast to the intermolecular, anti‐parallel β‐sheet composed of β‐strands from both RAS and effector RBDs [29]. The RHOG:ELMO2^RBD^ interface is mediated by electrostatic hydrogen bonds involving highly conserved residues, including Arg66 of RHOG which is solvent exposed in all other RAC GTPase structures, complemented by extensive hydrophobic interactions. Docking RHOG onto ELMO1^RDB^ in the context of the closed auto‐inhibited state of DOCK2:ELMO1 indicated that DOCK2^DHR2^ sterically occludes RHOG binding. Thus, RHOG engagement by ELMO1^RBD^ would promote the open‐active conformation of DOCK2:ELMO1. This explains how RHOG activates RAC1 by direct interaction with ELMO1^RBD^ *in vivo* in the context of the plasma membrane [52]. We note that, so far, this has been difficult to demonstrate *in vitro* with the purified ELMO1:DOCK2 complex, possibly because other factors, including intact membranes, may be required.

In the auto‐inhibited state of DOCK2:ELMO1, the binding site of ELMO^EID^ for the C‐terminal helix of the GPCR BAI1 (modelled on the ELMO2:BAI1 structure [59]) is sterically occluded by ELMO1^PH^ [29] (Fig. 8A), precluding BAI1 engagement. By contrast, in the open conformation, the BAI1‐binding site is accessible. Thus, similar to RHOG binding to ELMO1^RBD^, engagement of BAI1 to ELMO1^EID^ is only possible in the open, active conformation of DOCK2:ELMO1, thereby explaining how GPCR BAI1 stimulates DOCK2 GEF activity. An overlay of the auto‐inhibited and active states of DOCK2:ELMO1 is shown in Fig. 8C.

Regulation of DOCK2:ELMO1 through multiple‐phosphorylation events can also be explained from the auto‐inhibited state of DOCK2:ELMO1. TAM kinase‐dependent phosphorylation of Tyr18 of ELMO1^RBD^ promotes RAC activation and cell migration [60]. Phosphorylation of ELMO1^RBD^ on this Tyr residue would disrupt the ELMO1^RBD^–DOCK2^DHR2^ interface present in the closed auto‐inhibited binary DOCK2:ELMO1 complex, thereby relieving auto‐inhibition. A second phospho‐regulatory site in DOCK2:ELMO1 is a phosphorylation linker immediately N‐terminal of DOCK2^DHR3‐C2^ (Figs 1A, 7A and 8A). In the auto‐inhibited DOCK2:ELMO1 state, the phosphorylation linker of DOCK2 forms intermolecular interactions with ELMO1. Phosphorylation of three proximal residues within this segment (Tyr209, Tyr212 and Ser213, identified in PhosphoSitePlus) would destabilize the auto‐inhibited ELMO1 conformation. Consistent with this proposal, replacing these three residues in a phospho‐mimetic DOCK2 Glu mutant enhanced RAC1 activation, and cell migration and invasion [29].

## Architecture of the DOCK‐D subfamily indicates conserved structural features in the DOCK family

**Fig. 9:**
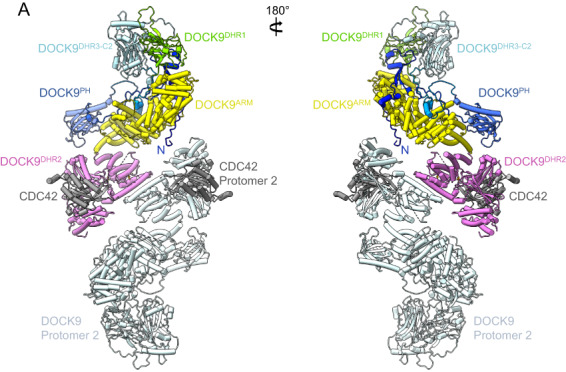
Model of the dimeric DOCK9:CDC42 complex. The model was based on the alphafold2 prediction of human DOCK9 [49] and the crystal structure of DOCK9^DHR2^:CDC42 [33] (PDB: 2WM9).

Full‐length structures of DOCK proteins from the DOCK‐C and DOCK‐D subfamilies have not yet been determined. However, alphafold2 predictions [49] of the monomeric states of DOCK9 and DOCK10, combined with crystal structures of the dimeric DOCK9^DHR2^ [33] and DOCK10^DHR2^ [43] proteins, enable the generation of models of full‐length dimeric DOCK9 and DOCK10. The alphafold2 models of DOCK9 and DOCK10 are predicted with high confidence [49]. Similar to that revealed from the cryo‐EM structures of DOCK2 and DOCK5, DOCK9 and DOCK10 are predicted to possess a DHR3‐C2 domain, that together with the DHR1 domain, form a pair of tandem C2 domains. All 11 human DOCK proteins share this DHR3‐C2 domain which is also conserved in the plant DOCK‐GEF SPIK1, for which a high‐confidence alphafold2 model was predicted [51]. The DHR3‐C2 domain in plants was found to have a complex lipid‐binding selectivity for both phospholipid headgroups and fatty acid chain saturation [51]. Also, similar to DOCK2 and DOCK5, the DHR1 and DHR2 domains of the DOCK‐D subfamily are separated by a long α‐solenoid segment (DOCK9^ARM^; Fig. 9). Uniquely, DOCK‐D subfamily proteins possess an N‐terminal PH domain. DOCK9^PH^ reportedly binds monophosphorylated inositides and localizes to membranes [61], however, compared with canonical PH domains [62], the inositide‐binding site in DOCK9^PH^ is not conserved. In the full‐length structures of DOCK9 and DOCK10, their hook‐like architecture positions the PH domain in close proximity to the DHR2 domain. Thus, it is possible that in DOCK‐D proteins, DOCK^PH^ might function similarly to ELMO1^PH^ of the DOCK‐A and DOCK‐B subfamilies, contacting the GTPase bound to DOCK^DHR2^ to either enhance GEF activity and/or contribute towards GTPase specificity. An ~ 100 residue segment N‐terminal to DOCK9^PH^ is predicted to form an extended structure, containing regions of local secondary structure that interacts along the length of DOCK9^ARM^ (Fig. 9).

The N‐terminal domains of DOCK6 and DOCK9 suppress GEF catalytic activity [11, 61, 63], suggestive of an auto‐inhibitory control mechanism in common with DOCK2:ELMO1. However, the alpahfold2‐derived models of DOCK6 and DOCK9 do not predict DHR2 domain interactions with N‐terminal segments, thus the molecular mechanisms for potential auto‐inhibition in DOCK6 and DOCK9 are yet to be defined.

## Conclusions

DOCK proteins represent archetypal signalling molecules composed of modular domains. There is a body of literature suggesting that an evolutionarily conserved interaction between DOCK1 and its orthologues in worm and fly interacts with the adapter proteins of the CRK family [13]. CRK proteins bind to DOCK1 C‐terminal proline‐rich regions and provide a signal to activate RAC, as assessed by genetic and cellular assays. In the ELMO1:DOCK2 and ELMO1:DOCK5 complexes described recently, the CRK‐binding regions are not resolved. Recently, *CRK‐Like* (*CRKL*) was found to be amplified in acral melanoma, where its protein product is bound to DOCK1 and DOCK5 [64]. Additional structural studies between ELMO:DOCK complexes and CRK adapters may reveal novel mechanisms of activation of these GEFs that could be targetable in anti‐cancer efforts.
